# Influence of Hyperglycemia on Dexmedetomidine-Induced Cardioprotection in the Isolated Perfused Rat Heart

**DOI:** 10.3390/jcm9051445

**Published:** 2020-05-13

**Authors:** Carolin Torregroza, Katharina Feige, Laura Schneider, Sebastian Bunte, Martin Stroethoff, André Heinen, Markus W. Hollmann, Ragnar Huhn, Annika Raupach

**Affiliations:** 1 Department of Anesthesiology, University Hospital Duesseldorf, Moorenstr. 5, 40225 Duesseldorf, Germany; Carolin.Torregroza@med.uni-duesseldorf.de (C.T.); Laura.Schneider@hhu.de (L.S.); Martin.Stroethoff@med.uni-duesseldorf.de (M.S.); Ragnar.Huhn@med.uni-duesseldorf.de (R.H.); Annika.Raupach@med.uni-duesseldorf.de (A.R.); 2 Department of Anesthesiology, Amsterdam University Medical Center (AUMC), Location AMC, Meiberdreef 9, 1105 AZ Amsterdam, The Netherlands; M.W.Hollmann@amsterdamumc.nl; 3 Department of Internal Medicine, Elbe Clinics Stade-Buxtehude, Bremervoerder Str. 111, 21682 Stade, Germany; buntesebastian@gmail.com; 4 Institute of Cardiovascular Physiology, Heinrich-Heine-University Duesseldorf, Universitaetsstr. 1, 40225 Duesseldorf, Germany; Andre.Heinen@hhu.de

**Keywords:** dexmedetomidine, cardioprotection, hyperglycemia, mannitol, ischemia/reperfusion

## Abstract

Pharmacological preconditioning (PC) and postconditioning (PoC), for example, by treatment with the α2-adrenoreceptor agonist Dexmedetomidine (Dex), protects hearts from ischemia-reperfusion (I/R) injury in experimental studies, however, translation into the clinical setting has been challenging. Acute hyperglycemia adversely affects the outcome of patients with myocardial infarction. Additionally, it also blocks cardioprotection by multiple pharmacological agents. Therefore, we investigated the possible influence of acute hyperglycemia on Dexmedetomidine-induced pre- and postconditioning. Experiments were performed on the hearts of male Wistar rats, which were randomized into 7 groups, placed in an isolated Langendorff system and perfused with Krebs-Henseleit buffer. All hearts underwent 33 min of global ischemia, followed by 60 min of reperfusion. Control (Con) hearts received Krebs-Henseleit buffer (Con KHB), glucose (Con HG) or mannitol (Con NG) as vehicle only. Hearts exposed to hyperglycemia (HG) received KHB, containing 11 mmol/L glucose (an elevated, but commonly used glucose concentration for Langendorff perfused hearts) resulting in a total concentration of 22 mmol/L glucose throughout the whole experiment. To ensure comparable osmolarity with HG conditions, normoglycemic (NG) hearts received mannitol in addition to KHB. Hearts were treated with 3 nM Dexmedetomidine (Dex) before (DexPC) or after ischemia (DexPoC), under hyperglycemic or normoglycemic conditions. Infarct size was determined by triphenyltetrazoliumchloride staining. Acute hyperglycemia had no impact on infarct size compared to the control group with KHB (Con HG: 56 ± 9% ns vs. Con KHB: 56 ± 7%). DexPC reduced infarct size despite elevated glucose levels (DexPC HG: 35 ± 3%, *p* < 0.05 vs. Con HG). However, treatment with Dex during reperfusion showed no infarct size reduction under hyperglycemic conditions (DexPoC HG: 57 ± 9%, ns vs. Con HG). In contrast, hearts treated with mannitol demonstrated a significant decrease in infarct size compared to the control group (Con NG: 37 ± 3%, *p* < 0.05 vs. Con KHB). The combination of Dex and mannitol presents exactly opposite results to hearts treated with hyperglycemia. While DexPC completely abrogates infarct reduction through mannitol treatment (DexPC NG: 55 ± 7%, *p* < 0.05 vs. Con NG), DexPoC had no impact on mannitol-induced infarct size reduction (DexPoC NG: 38 ± 4%, ns vs. Con NG). Acute hyperglycemia inhibits DexPoC, while it has no impact on DexPC. Treatment with mannitol induces cardioprotection. Application of Dex during reperfusion does not influence mannitol-induced infarct size reduction, however, administering Dex before ischemia interferes with mannitol-induced cardioprotection.

## 1. Introduction

Pharmacological preconditioning (PC) and postconditioning (PoC) by treatment with several different substances, e.g., volatile anesthetics, opioids or phosphodiesterase-inhibitors, have been proven to protect hearts from ischemia-reperfusion (I/R) injury and lead to significant infarct size reduction in experimental studies, comparable to results from ischemic preconditioning [1]. However, recent clinical trials that attempted to translate conditioning strategies into a clinical setting have been disappointing [2,3,4,5]. Besides the possible influence of different anesthetics on cardioprotection, e.g., propofol, the comorbidities of patients suffering myocardial infarction and I/R injury have been found to be a challenging factor in the translation of conditioning strategies into the clinical setting [6,7]. Acute hyperglycemia plays an important role in adverse outcomes in patients with and without diabetes after myocardial infarction [8,9]. Elevated glucose levels at the time of admission have been shown to correlate with increased mortality and morbidity in these patients [10]. Several previous studies have described the influence of acute hyperglycemia on ischemic [11,12] and pharmacological [13,14] preconditioning, with both stimuli blocking cardioprotection.

The highly selective α2-adrenoreceptor agonist Dexmedetomidine (Dex), which has sedative, analgesic and opioid-sparing effects, is routinely used in the perioperative setting, especially for short- as well as long-term sedation in intensive care patients [15]. Previously, we [16,17], and others [18], have demonstrated the cardioprotective effects of Dexmedetomidine-induced pre- and postconditioning in an in vitro as well as an in vivo I/R injury model in the rat heart. Dexmedetomidine protects the heart through its direct effects on myocardial signaling cascades [19], e.g., activation of the reperfusion injury salvage kinase (RISK), and in particular, the phosphatidylinositol-3 kinase/protein kinase B (PI3K/Akt) [18] signaling pathway. Furthermore, it has been shown that Dexmedetomidine-induced cardioprotection is mediated by mitochondrial large-conductance Ca^2+^-sensitive potassium (mBK_Ca_) [16] and ATP-sensitive potassium (mK_ATP_)-channels [20]. As shown in several experimental studies, Dexmedetomidine has the ability to significantly reduce infarct size after I/R injury, and in fact it is already routinely used in patients, thus, it is a highly promising pharmacological target for translating conditioning strategies into the clinical setting.

The interference of signaling cascades involved in cardioprotection during disease states, such as diabetes mellitus, has previously been identified. Diabetes mellitus, for instance, prevents ischemic preconditioning in patients suffering from an acute myocardial infarction [21]. Ishihara et al. found that the presence of prodromal angina, as an equivalent of ischemic preconditioning, seemed to ameliorate outcome in patients without diabetes mellitus, while patients suffering from this comorbidity did not show a reduction in mortality [21]. While some studies have suggested a cardioprotective effect of Dexmedetomidine-induced postconditioning in rats with Type II diabetes mellitus (DM II) [18,22] and I/R injury, the influence of acute hyperglycemia on Dexmedetomidine-induced cardioprotection has not been investigated so far. Hyperglycemia is one of the most relevant influencing factors in patients with I/R injury, as it is often part of chronic metabolic comorbidities such as diabetes mellitus in these patients. Furthermore, hyperglycemia also occurs frequently when the human body is exposed to any kind of stressful condition [23], like myocardial infarction, due to the activation of the sympathetic nervous system.

Therefore, with this study we set out to determine whether acute hyperglycemia has a potential impact on Dexmedetomidine-induced pre- and/or postconditioning in an in vitro I/R rat heart model.

## 2. Material and Methods

The present study conforms to the Guide for the Care and Use of Laboratory Animals published by the U.S. National Institute of Health (NIH publication No. 85–23, revised 1996) and was approved by the local Animal Care and Use Committee of the University of Duesseldorf (Project number O27/12). The animals were obtained from the breeding facility at the Central Animal Research Facility of the Heinrich-Heine-University Duesseldorf.

### 2.1. Surgical Preparation and Langendorff Model

All experiments were performed on male Wistar rats (2–3 months old), which were randomized into seven groups. Animals were anesthetized with intraperitoneal injection of pentobarbital (80 mg/kg body weight, Narcoren, Merial, Germany) and decapitated. Hearts were excised through a thoracotomy, placed onto a Langendorff system under constant pressure (80 mmHg) and temperature (37 °C) and perfused with Krebs-Henseleit buffer (118 mM NaCl, 4.7 mM KCl, 1.2 mM MgSO_4_, 1.17 mM KH_2_PO_4_, 24.9 mM NaHCO_3_, 2.52 mM CaCl_2_, 11 mM glucose, and 1 mM lactate) enriched with a mix of 95% O_2_ and 5% CO_2_. The procedure was performed as described previously [24]. A saline-filled balloon was inserted into the left ventricle and the end-diastolic pressure set to 4–6 mmHg for continuous pressure measurements. Hemodynamic data was measured continuously, digitized using an analogue to digital converter (PowerLab/8SP, ADInstruments Pty Ltd., Castle Hill, Australia) at a sampling rate of 500 Hz and recorded on a personal computer using Labchart 8.0 for Windows (ADInstruments Pty Ltd., Castle Hill, Australia). Hemodynamic data included heart rate, left ventricular end-systolic pressure (LVESP), left ventricular end-diastolic pressure (LVEDP) and left ventricular developed pressure (LVDP) (calculated as LVESP-LVEDP), maximal rate of rise of left ventricular pressure (dP/dt max.) as well as coronary flow. Furthermore, maximal contracture during ischemia and the respective time-point as well as the time to ischemic contracture was analyzed for each experiment as an indicator for myocardial injury. Glucose levels (mmol/L) were measured after collecting effluent from each heart at baseline (minute 10), after the start of glucose or mannitol treatment but before PC (minute 19) and during reperfusion (minute 122).

At the end of reperfusion, hearts were cut into 8 transverse slices per heart (2 mm each slice) and stained with 0.75% triphenyltetrazoliumchloride (TTC) solution. The size of the infarcted area was determined by planimetry using SigmaScan Pro5 software by a blinded, experienced investigator [25]. Infarct size was expressed as percentage of infarct area per total area of the left ventricle.

### 2.2. Experimental Setting

**Figure 1:**
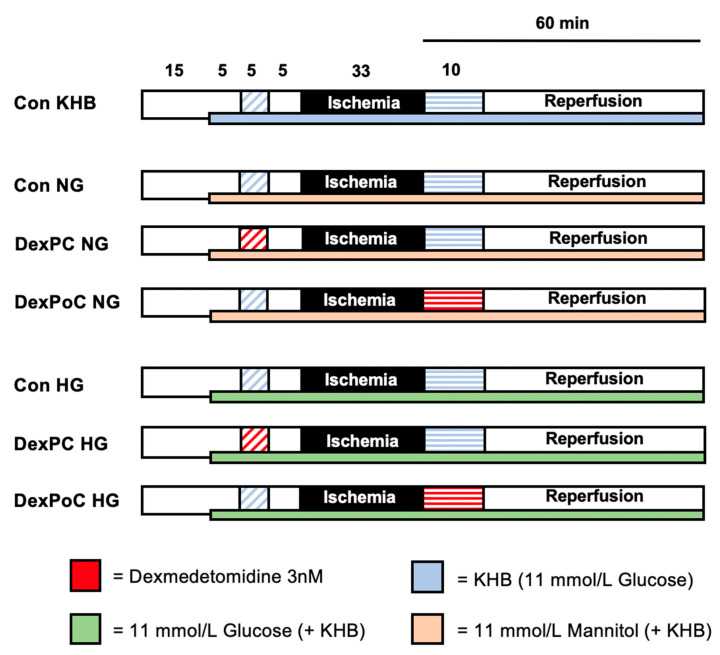
Experimental protocol. Con = Control; KHB = Krebs-Henseleit buffer, NG = Normoglycemia, HG = Hyperglycemia; Dex = Dexmedetomidine; PC = Preconditioning; PoC = Postconditioning.

A total of 50 rat hearts were randomized into 7 experimental groups (n = 6–8 per group). All hearts underwent 15 min of adaption period, 15 min of the first treatment phase and 33 min of ischemia, followed by 60 min of reperfusion, including the second treatment phase. The first subgroup consisted of hearts under hyperglycemic (HG) conditions. The second subgroup included the same treatment phases but represents a normoglycemic (NG) state under comparable osmolar conditions to hyperglycemia groups. To ensure comparable osmolarity in all subgroups, the hearts in the second subgroup received mannitol treatment. The Krebs-Henseleit buffer used for this protocol already consists of 11 mmol/L glucose. For normoglycemic conditions, 11 mmol/L glucose was administered, as this is the most commonly used concentration in the Langendorff model of the isolated perfused heart [26]. Furthermore, postprandial blood glucose levels in Wistar rats can increase up to 10.4 mmol/L [27]. To achieve hyperglycemia, hearts in the hyperglycemic subgroup were perfused with a total of 22 mmol/L glucose (11 mmol/L from the KHB plus additional glucose solution with a final concentration of 11 mmol/L glucose in the heart). Thus, in return, normoglycemic hearts received 11 mmol/L mannitol treatment (final concentration in the heart) in addition to KHB. To establish hyperglycemic or normoglycemic conditions before Dexmedetomidine treatment, perfusion with additional glucose or mannitol was started 5 min prior to the preconditioning treatment phase (Figure 1).

Control (Con KHB): Hearts received Krebs-Henseleit buffer (KHB) as vehicle during both treatment phases (pre- and postconditioning) at an infusion rate of 1% coronary flow (CF).

Dexmedetomidine (Dex): For treatment we used 3 nM Dexmedetomidine during pre- and postconditioning. This is the previously determined lowest concentration inducing the strongest cardioprotective effect [16,17].

Preconditioning (PC): Hearts were treated with 3 nM Dexmedetomidine over 5 min, followed by a 5-min wash-out period before 33 min of ischemia. In previous studies, we demonstrated that treatment with 3 nM Dexmedetomidine for 5 min followed by a 5-min wash-out phase before ischemia induces a strong cardioprotective effect and significant reduction in infarct size [16]. A longer period of preconditioning or higher concentrations of Dexmedetomidine were not able to reduce the infarct size to a greater extent [16].

Postconditioning (PoC): Hearts were treated with 3 nM Dexmedetomidine over 10 min, immediately after ischemia during reperfusion. As for postconditioning with Dexmedetomidine, previous investigations have shown that infarct size reduction by treatment with 3 nM Dexmedetomidine is completely independent of time point or duration during reperfusion [17]. Based on these findings and others [28] we chose a 10-min postconditioning treatment immediately at the onset of reperfusion.

#### 2.2.1. Subgroup—Normoglycemia (NG)

After 15 min of adaption period, all hearts were perfused with a mannitol solution in addition to KHB (containing 11 mol/L glucose) at an infusion rate of 1% CF to achieve a total final concentration of 11 mmol/L mannitol resulting in 22 mmol/L monosaccharides (glucose and mannitol) in the heart.

Control (Con NG): Hearts were perfused with KHB as vehicle during pre- and postconditioning and received mannitol throughout the whole experiment.

DexPC NG: Preconditioning treatment with 3 nM Dexmedetomidine for 5 min under normoglycemia.

DexPoC NG: Postconditioning treatment with 3 nM Dexmedetomidine for 10 min under normoglycemia.

#### 2.2.2. Subgroup—Hyperglycemia (HG)

After 15 min of adaption period, all hearts were perfused with a glucose solution in addition to KHB (containing 11 mol/L glucose) at an infusion rate of 1% CF, resulting in a total of 22 mmol/L glucose final concentration in the heart.

Control (Con HG): Hearts received KHB as vehicle during both treatment phases as well as a total of 22 mmol/L glucose during the whole experiment.

DexPC HG: Preconditioning treatment with 3 nM Dexmedetomidine for 5 min under hyperglycemia.

DexPoC HG: Postconditioning treatment with 3 nM Dexmedetomidine for 10 min under hyperglycemia.

### 2.3. Statistical Analysis

Sample size calculation (GraphPad StatMate™, GraphPad Software, San Diego, CA, USA) suggested a group size of n = 7 for detecting a 25% mean difference and a standard deviation of 15% in infarct size (power 80%, α < 0.05 (two-tailed)). Infarct sizes were analyzed by one-way analysis of variance (ANOVA) and a Tukey’s post hoc test. We performed a two-way ANOVA and a Tukey post hoc test (GraphPad Software V7.01, San Diego, CA, USA) for comparison of hemodynamic data and glucose levels between groups as well as between different time points within groups. Data is presented as mean ± standard deviation (SD). *p* < 0.05 was considered statistically significant for changes within and between groups.

## 3. Results

### 3.1. Animal Characteristics

As shown in Table 1, there were no significant differences in body weight, wet weight and the time and level of maximal ischemic contracture between and within all groups.

### 3.2. Infarct Size Measurements

**Figure 2:**
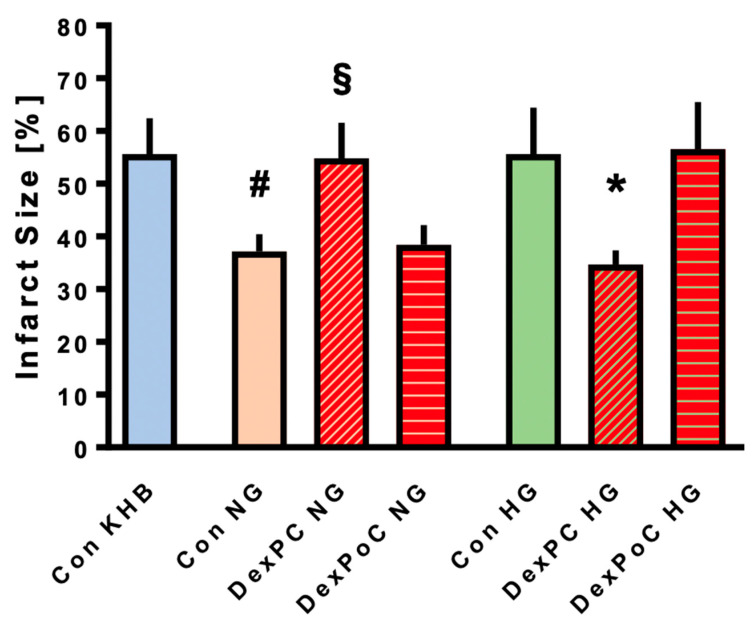
Infarct size measurements. Figure shows the infarct size of controls (Con) and Dexmedetomidine (Dex) treated groups under hyperglycemia (HG) or normoglycemia (NG). PC = Preconditioning, PoC = Postconditioning. Data are presented as means ± SD, * *p* < 0.05 vs. Con HG and DexPoC HG, respectively, ^#^ *p* < 0.05 vs. Con KHB, ^§^ *p* < 0.05 vs. Con NG and DexPoC NG, respectively.

Results from the infarct size measurements are shown in Figure 2. The infarct size in control (Con) hearts, which were only treated with Krebs-Henseleit Buffer (KHB) as vehicle, was 56 ± 7%. Acute hyperglycemia had no impact on infarct size compared to the control group with KHB (Con HG: 56 ± 9% ns vs. Con KHB). Dexmedetomidine-induced preconditioning (DexPC HG) significantly reduced infarct size to 35 ± 3%, despite elevated glucose levels (*p* < 0.0001 vs. Con HG). However, treatment with Dexmedetomidine during reperfusion (DexPoC) showed no infarct size reduction under hyperglycemic conditions (DexPoC HG: 57 ± 9%, ns vs. Con HG). In contrast to HG conditions, control hearts treated with mannitol only (Con NG) demonstrated a significant decrease in infarct size to 37 ± 3% compared to the KHB control group (*p* = 0.0001 vs. Con KHB). As for the combination of Dexmedetomidine and mannitol, results show the exact opposite to hearts treated with hyperglycemia. While Dexmedetomidine-induced preconditioning (DexPC) completely abrogates reduction of infarct size by mannitol treatment (DexPC NG: 55 ± 7%, *p* = 0.0003 vs. Con NG), DexPoC had no impact on mannitol-induced infarct size reduction (DexPoC NG: 38 ± 4%, ns vs. Con NG).

### 3.3. Cardiac Function

Hemodynamic data for all groups are shown in Table 2. There were no differences measured for heart rate and coronary flow between groups. Hearts in the control KHB group had a significantly lower left ventricular developed pressure (LVDP) before ischemia compared to control hearts in the two subgroups (Con NG and Con HG). However, there was no difference in LVDP between hearts in hyperglycemia and normoglycemia subgroups. As expected, LVDP and dP/dt max. significantly decreased while LVEDP increased during reperfusion compared to baseline in all groups. Furthermore, coronary flow decreased significantly during reperfusion compared to baseline with the exception of the Con KHB and Con NG groups.

### 3.4. Glucose Values

Glucose values (mmol/L) for all groups are reported in Table 3. No differences were measured between all groups under baseline conditions and within KHB and NG groups throughout the whole experiment. Hearts under hyperglycemic condition had significantly higher glucose levels after starting glucose treatment (pretreatment (PT), minute 19, before initiation of Dexmedetomidine preconditioning) and at the end of reperfusion (minute 122) compared to hearts in the normoglycemia subgroups and Con KHB group. Furthermore, glucose levels were also significantly elevated before ischemia (PT) and during reperfusion compared to baseline (before administration of glucose, minute 10) within each hyperglycemia group. There were no statistically significant differences in glucose values between PT and reperfusion within any subgroup.

## 4. Discussion

The results from the present study show that acute hyperglycemia completely inhibits cardioprotection by Dexmedetomidine-induced postconditioning whereas it has no impact on preconditioning with Dexmedetomidine. Furthermore, based on our findings we were able to demonstrate that treatment with mannitol has a profound cardioprotective effect by reducing infarct size after I/R injury in the isolated rat heart. Interestingly, administration of Dexmedetomidine before ischemia interferes with mannitol-induced cardioprotection, while application of Dexmedetomidine during reperfusion has no influence on infarct size reduction by mannitol.

Although we see a strong effect on infarct size, we did not detect hemodynamic improvement during the reperfusion phase among the groups. The exact reason for this is unclear, but it might be due to the occurrence of myocardial stunning, i.e., a temporary depression of cardiac function in the surviving myocardial tissue, which can last for several days after I/R injury [29]. Furthermore, while contractile dysfunction possibly reflects ischemic injury it is not synonymous with cell death [30]. For our study, we chose to assess quantitative cardiomyocyte viability through infarct size measurement by TTC staining, which is still considered the gold standard for detecting infarct reducing effects in the Langendorff model of isolated reperfused hearts.

### 4.1. Dexmedetomidine and Hyperglycemia

Acute hyperglycemia is a major factor in patients suffering myocardial infarction and plays a crucial role in cardiovascular morbidity and mortality after I/R injury [31]. Previous studies have shown a significant correlation between blood glucose level and long-term outcome in patients with acute myocardial infarction, independent of diabetes mellitus as a comorbidity [8,10]. While there have been few studies regarding Dexmedetomidine-induced cardioprotection and diabetes mellitus, the influence of hyperglycemia on this highly selective alpha-2-adrenoreceptor agonist has not been investigated so far.

Cheng et al. 2018 [18] showed that in an in vivo rat model, Dexmedetomidine-induced postconditioning maintains its cardioprotective properties despite hyperglycemia due to type II diabetes. However, in this study rats received diabetes-inducing treatment for 4 weeks prior to the experiments. Thus, this study focused on the influence of diabetes and not acute hyperglycemia on Dexmedetomidine treatment in the context of cardiac function after I/R injury.

Hyperglycemia has been shown to interfere with different kinds of cardioprotective stimuli, e.g., ischemic preconditioning (IPC) [11,31,32] and remote ischemic perconditioning (RIPerC) [33]. In the study by Baranyai et al. [34], infarct size reduction induced by RIPerC was completely blocked under hyperglycemic conditions, presumably by interfering with activation of the mechanistic Target of Rapamycin (mTOR) pathway and Akt phosphorylation as well as elevation of nitric oxide (NO) and autophagy. However, acute hyperglycemia per se seems to have no influence on infarct size. 

The mechanism of hyperglycemia that eradicates cardioprotective strategies is not fully understood. IPC, for example, reduces accumulation of polymorphonuclear granulocytes (PMNs) and this effect is reversed under hyperglycemic conditions [35]. Furthermore, IPC is linked to an increase in myocardial glucose uptake and hyperglycemia seems to interfere with these changes during reperfusion [11]. 

Pharmacologically-induced cardioprotection has been a promising, less invasive strategy and a major research focus in recent years. However, similar to IPC or RIPerC, several studies have shown that acute hyperglycemia and diabetes mellitus have a significant impact on these conditioning strategies [13,36,37]. Isoflurane-induced preconditioning is fully blocked by hyperglycemia [13] due to inactivation of mK_ATP_ channels [38,39]. Isoflurane-induced postconditioning leads to a significant reduction in infarct size and creatine kinase myocardial band (CK-MB) levels in experimental studies [40]. Furthermore, administration of the volatile anesthetic increases expression of phosphorylated Akt and endothelial nitric oxide synthase (eNOS) after I/R injury. All these cardioprotective effects are completely abolished under elevated glucose levels [40]. Both Akt, as part of the RISK pathway, and eNOS are involved in signaling cascades of different preconditioning stimuli [41]. Activation of the RISK pathway eventually results in the inhibition of the mitochondrial permeability transition pore (MPTP) opening during reperfusion, thereby preventing cell damage and cell death. In a previous study on the influence of hyperglycemia on Sevoflurane-induced cardioprotection, inhibition of MPTP with Cyclosporin A (CsA) reversed the loss of cardioprotection that occurred during hyperglycemic treatment [37]. These results suggest that regulation of MPTP might be a crucial step in the interaction of hyperglycemia and cardioprotective mechanisms. 

Phosphorylation of Akt and increasing levels of eNOS play a pivotal role in Dexmedetomidine-induced cardioprotection [18,19,42]. eNOS is known to activate soluble guanylate cyclase (sGC), which in turn increases cyclic guanosine monophosphate (cGMP) and finally results in activation of protein kinase G (PKG) [41]. The mitochondrial K_ATP_ channel is the target of both NO and PKG. The influence of acute hyperglycemia on these channels is highly relevant considering the results of our study, as Dexmedetomidine-induced pre- and postconditioning is mediated via activation of mK_ATP_ channels [20,43]. Therefore, acute hyperglycemia might have an impact on Dexmedetomidine-induced cardioprotection by influencing the RISK pathway [44,45,46,47] and/or NO/PKG pathway. Besides the possible influence of hyperglycemia on the aforementioned signaling cascades, it also seems to affect levels of reactive oxygen species (ROS), and thereby, pharmacologically-induced cardioprotection. Kehl et al. showed that an excessive amount of ROS is generated under hyperglycemic conditions [13,48], which in turn blocks Isoflurane-induced preconditioning [49]. While release of small amounts of ROS, e.g., due to opening of mK_ATP_ channels, is indeed involved in cardioprotection [50,51,52], it has been shown that excessive amounts of ROS leads to opening of the MPTP, which ultimately results in cell damage and death.

The striking difference in the influence of hyperglycemia on Dexmedetomidine pre- and postconditioning might be partly explained by the administration time of glucose in our study. Although we started the application of glucose before preconditioning with Dexmedetomidine and glucose levels were already significantly increased at this time, the impact of hyperglycemia on RISK and eNOS/PKG pathways as well as ROS formation was potentially not as pronounced at this point and therefore Dexmedetomidine-induced preconditioning was still effective. At the time of reperfusion, hearts were exposed to hyperglycemic conditions for 48 min, in contrast to only 5 min before Dexmedetomidine-preconditioning. This prolonged period of hyperglycemia probably completely prevented cardioprotection by Dexmedetomidine-induced postconditioning. In order to clarify this issue, further studies regarding the length as well as the time point of administration of glucose in combination with Dexmedetomidine are necessary.

### 4.2. Dexmedetomidine and Mannitol

Interestingly, results from our study demonstrate that treatment with mannitol induces a strong cardioprotective effect through infarct size reduction. However, to this point, little research has been published on hyperosmolarity and conditioning strategies [32].

Chen at al. [53] demonstrated a connection between hyperosmolarity and type I diabetes mellitus (DMI) with regard to cardioprotection in isolated rat hearts. Hearts from DM1 rats had significantly better post-ischemic cardiac function after I/R injury, combined with higher levels of the—with osmolarity associated—heat shock protein 90 (hsp90). Improvement in post-ischemic cardiac function was halted by additional treatment with an hsp90 inhibitor. Hearts treated with mannitol also showed larger amounts of hsp90, similar to those of DMI hearts. These results suggest that hyperosmolarity induced by mannitol treatment might improve cardiac function after I/R injury via hsp90. In contrast, Chiong at al. [54] showed that sorbitol-induced hyperosmolarity resulted in increased calcium concentration in cardiomyocytes and along with that, pronounced apoptosis. Presumably, an increase in influx into the cell through voltage-dependent L-type Ca^2+^-channels as well as an elevated release of calcium from the sarcoplasmatic reticulum is involved in this. However, we were able to show that mannitol, despite the influence of hyperosmolarity on calcium homeostasis, was able to induce a strong cardioprotective effect. As suggested by Zálešák et al. [55], conditioning strategies by mannitol treatment might be dependent on the balance between calcium homeostasis and activation of cardioprotective signaling cascades, such as the RISK-pathway. However, further studies are necessary to fully understand the mechanisms of cardioprotection induced by mannitol.

Results from our study indicate that administering Dexmedetomidine before ischemia interferes with mannitol-induced cardioprotection. These findings are in line with results from Zálešák et al. [55] on hyperosmotic environment and IPC. Similar to our results, they showed a cardioprotective effect of mannitol treatment that was completely abrogated in combination with IPC. Along with an increase in infarct size when combining mannitol and IPC, levels of heart-type fatty acid binding protein (h-FABP) as a biomarker for cell injury were also higher in this group compared to hearts with IPC treatment only. Dexmedetomidine-induced preconditioning is fairly similar to IPC in a lot of aspects, e.g., involvement of signaling cascades. Both ischemic and Dexmedetomidine-induced preconditioning is mediated, among others, via the RISK pathway, which is also activated by hyperosmotic conditions [55,56]. One possible explanation for these results might be a counteracting effect of mannitol and Dexmedetomidine regarding the cardioprotective signaling pathways, which could lead to a predominantly negative impact of hyperosmolarity on cardiac function, due to intracellular calcium overload and oscillation during reperfusion and along with that, increased apoptosis. However, at this point we can only speculate, and further experiments are needed.

Interestingly, application of Dexmedetomidine during reperfusion did not influence mannitol-induced infarct size reduction in our study. In accordance with our results are the findings of Baranvai et al. [34] regarding RIPerC and mannitol treatment in an in vivo rat model. RIPerC under hyperosmotic conditions showed significantly lower infarct sizes than in hearts treated with mannitol only. However, in contrast to our study, they were not able to show an infarct size reducing effect of mannitol itself. Nevertheless, these findings at least suggest that application of a perconditioning stimulus and mannitol treatment do not counteract each other, as seems to be the case under preconditioning and hyperosmolarity.

### 4.3. Limitations

As for most studies, there are some limitations that need to be addressed. The used concentration of 11 mmol/L glucose to mimic normoglycemia does not represent the physiological condition for rats, which is typically about 5–7 mmol/L glucose measured in the blood [35]. Notably, postprandial blood glucose in Wistar rats can indeed increase up to 10.4 mmol/L [27]. However, this concentration of 11 mmol/L glucose is most commonly used for Langendorff perfused hearts [26], thus increasing the comparability of this study with other studies investigating cardioprotective effects. Additionally, we have previously demonstrated an infarct size reducing effect of Dexmedetomidine pre- and postconditioning under perfusion with KHB containing 11 mmol/L glucose [16,17,57]. Along with the glucose concentration under normoglycemia, the treatment with 22 mmol/L glucose in the hyperglycemic groups was also chosen in conformity with the literature [11,34,58,59,60] and our own previous studies [35,36,37]. This is the typically used glucose concentration in studies investigating the influence of acute hyperglycemia on conditioning strategies.

We did not investigate possible underlying mechanisms or pathways mediating cardioprotection of Dexmedetomidine in this study. We acknowledge that this is an interesting and relevant question that could possibly be addressed in further follow-up studies. However, our focus in the present study was to unravel a possible effect of hyperglycemia or mannitol on Dexmedetomidine-induced conditioning strategies. This in vitro model was the first approach to this topic and should be verified in an in vivo study, before translation into the clinical setting might be conceivable.

In conclusion, the administration of Dexmedetomidine for cardioprotection in ischemia reperfusion injury is a promising clinical approach. Therefore, unravelling the potential influencing factors on this conditioning strategy, especially comorbidities like diabetes mellitus along with acute hyperglycemia, is highly relevant in the context of Dexmedetomidine-induced cardioprotection. Results from our study demonstrate that while peracute hyperglycemia before ischemia might not interfere with Dexmedetomidine-induced preconditioning, hyperglycemic conditions at the time of reperfusion seem to be critical in postconditioning with Dexmedetomidine. As treatment before myocardial ischemia is not predictable in most cases, postconditioning strategies are of high interest in the clinical scenario; therefore, this aspect should be included in future translational studies with Dexmedetomidine-induced postconditioning.

## Figures and Tables

**Table 1:** Weights and ischemic contracture.

Subgroup	Treatment	n	Body Weight (g)	Heart Wet Weight (g)	Time of Max. Ischemic Contracture (min)	Level of Max. Ischemic Contracture (mmHg)
KHB	Con	7	295 ± 14	1.17 ± 0.07	17 ± 3	107 ± 16
NG	Con	6	299 ± 17	1.23 ± 0.04	17 ± 1	76 ± 21
	DexPC	7	299 ± 15	1.25 ± 0.09	16 ± 2	70 ± 14
	DexPoC	8	308 ± 26	1.21 ± 0.05	16 ± 3	71 ± 17
HG	Con	8	301 ± 10	1.26 ± 0.04	15 ± 1	107 ± 26
	DexPC	6	309 ± 21	1.28 ± 0.03	17 ± 2	98 ± 16
	DexPoC	8	306 ± 19	1.25 ± 0.06	14 ± 2	102 ± 14

Data are mean ± SD; Con = Control; KHB = Krebs-Henseleit buffer; NG = Normoglycemia; HG = Hyperglycemia; Dex = Dexmedetomidine; PC = Preconditioning; PoC = Postconditioning.

**Table 2:** Hemodynamic variables.

Subgroup	Treatment	Baseline	PC	Reperfusion	
				30	60
Heart Rate (bpm)					
KHB	Con	319 ± 43	305 ± 48	260 ± 53	243 ± 50
NG	Con	286 ± 21	268 ± 16	317 ± 40	207 ± 66
	DexPC	308 ± 23	288 ± 20	287 ± 54	232 ± 54
	DexPoC	314 ± 40	293 ± 42	298 ± 47	250 ± 71
HG	Con	289 ± 41	286 ± 32	235 ± 71	209 ± 60
	DexPC	302 ± 26	286 ± 26	265 ± 99	202 ± 40
	DexPoC	305 ± 44	293 ± 36	254 ± 60	217 ± 33
LVDP (mmHg)					
KHB	Con	105 ± 14	110 ± 11	27 ± 16 *	33 ± 14 *
NG	Con	141 ± 31 ^#^	148 ± 28 ^#^	32 ± 15 *	43 ± 19 *
	DexPC	129 ± 36	129 ± 32	24 ± 19 *	30 ± 17 *
	DexPoC	136 ± 30	133 ± 34	34 ± 19 *	40 ± 18 *
HG	Con	141 ± 23 ^#^	145 ± 33 ^#^	20 ± 9 *	32 ± 12 *
	DexPC	118 ± 27	126 ± 29	20 ± 6 *	32 ± 6 *
	DexPoC	140 ± 38	145 ± 30	28 ± 18 *	36 ± 16 *
LVEDP (mmHg)					
KHB	Con	4 ± 2	4 ± 3	129 ± 19 *	107 ± 13 *
NG	Con	4 ± 1	3 ± 2	109 ± 28 *	94 ± 22 *
	DexPC	4 ± 1	4 ± 2	106 ± 14 *	93 ± 14 *
	DexPoC	5 ± 2	5 ± 2	99 ± 18 *	89 ± 18 *
HG	Con	4 ± 2	4 ± 2	127 ± 29 *	109 ± 19 *
	DexPC	6 ± 2	7 ± 3	129 ± 17 *	111 ± 16 *
	DexPoC	4 ± 1	4 ± 3	123 ± 20 *	112 ± 19 *
dP/dt max. (mmHg/s)					
KHB	Con	4357 ± 1047	4976 ± 1097	1256 ± 313 *	1699 ± 625 *
NG	Con	4793 ± 1337	5356 ± 1291	2559 ± 1429 *	2230 ± 1681 *
	DexPC	4470 ± 1119	4805 ± 1143	1588 ± 611 *	1603 ± 362 *
	DexPoC	4974 ± 953	5055 ± 1063	2351 ± 966 *	2093 ± 923 *
HG	Con	5312 ± 1299	5476 ± 859	1453 ± 310 *	1726 ± 481 *
	DexPC	4337 ± 1089	5004 ± 1347	2242 ± 1991 *	1461 ± 259 *
	DexPoC	4834 ± 1470	5190 ± 1489	1696 ± 791 *	1821 ± 1007 *
Coronary flow (mL/min)					
KHB	DexPoC	11 ± 2	10 ± 2	8 ± 1	9 ± 3
NG	Con	12 ± 2	11 ± 2	9 ± 2	9 ± 2
	DexPC	14 ± 4	14 ± 5	10 ± 2 *	9 ± 2 *
	DexPoC	15 ± 5	14 ± 6	10 ± 1 *	9 ± 3 *
HG	Con	13 ± 2	13 ± 3	8 ± 2 *	7 ± 2 *
	DexPC	12 ± 3	12 ± 3	7 ± 2 *	7 ± 2 *
	DexPoC	14 ± 4	14 ± 4	6 ± 1 *	6 ± 1 *

Data are mean ± SD; Con = Control; KHB = Krebs-Henseleit buffer; NG = Normoglycemia; HG = Hyperglycemia; Dex = Dexmedetomidine; PC = Preconditioning; PoC = Postconditioning; LVDP = Left Ventricular Developed Pressure; LVEDP = Left Ventricular End-diastolic Pressure; dP/dt max. = Maximal Rate of Rise of Left Ventricular Pressure * *p* < 0.05 versus Baseline; ^#^ *p* < 0.05 versus Con KHB.

**Table 3:** Glucose values (mmol/L).

Subgroup	Treatment	Baseline	PT	Reperfusion
KHB	Con	11.0 ± 0.2	10.7 ± 0.4	10.7 ± 0.2
NG	Con	11.1 ± 0.4	10.9 ± 0.3	10.9 ± 0.3
	DexPC	11.1 ± 0.3	10.9 ± 0.2	11.0 ± 0.5
	DexPoC	11.2 ± 0.2	11.1 ± 0.3	11.0 ± 0.2
HG	Con	11.0 ± 0.3	21.6 ± 0.5 *^,#^	23.1 ± 2.5 *^,#^
	DexPC	11.2 ± 0.3	22.1 ± 0.7 *^,§^	23.2 ± 1.1 *^,§^
	DexPoC	11.0 ± 0.3	22.0 ± 0.5 *^,$^	23.7 ± 0.8 *^,$^

Data are mean ± SD; Con = Control; KHB = Krebs-Henseleit buffer; NG = Normoglycemia; HG = Hyperglycemia; Dex = Dexmedetomidine; PT = Pretreatment; PC = Preconditioning; PoC = Postconditioning. * *p* < 0.05 versus Baseline, ^#^ *p* < 0.05 versus Con KHB and Con NG, ^§^ *p* < 0.05 versus DexPC NG, ^$^ *p* < 0.05 versus DexPoC NG.
